# Supplementary material for: Mechanism of ASF1 Inhibition by CDAN1
Source: bioRxiv. 2024 Aug 8:2024.08.08.607204. Preprint. [Version 1] doi: 10.1101/2024.08.08.607204 (PMC11326237; doi:10.1101/2024.08.08.607204)
Supplement: 1 [file NIHPP2024.08.08.607204v1-supplement-1.pdf]

bioRxiv preprint doi: https://doi.org/10.1101/2024.08.08.607204; this version posted August 8, 2024. The copyright holder for this preprint (which was not certified by peer review) is the author/funder, who has granted bioRxiv a license to display the preprint in perpetuity. It is made available under aCC-BY 4.0 International license.

Supplemental Figures

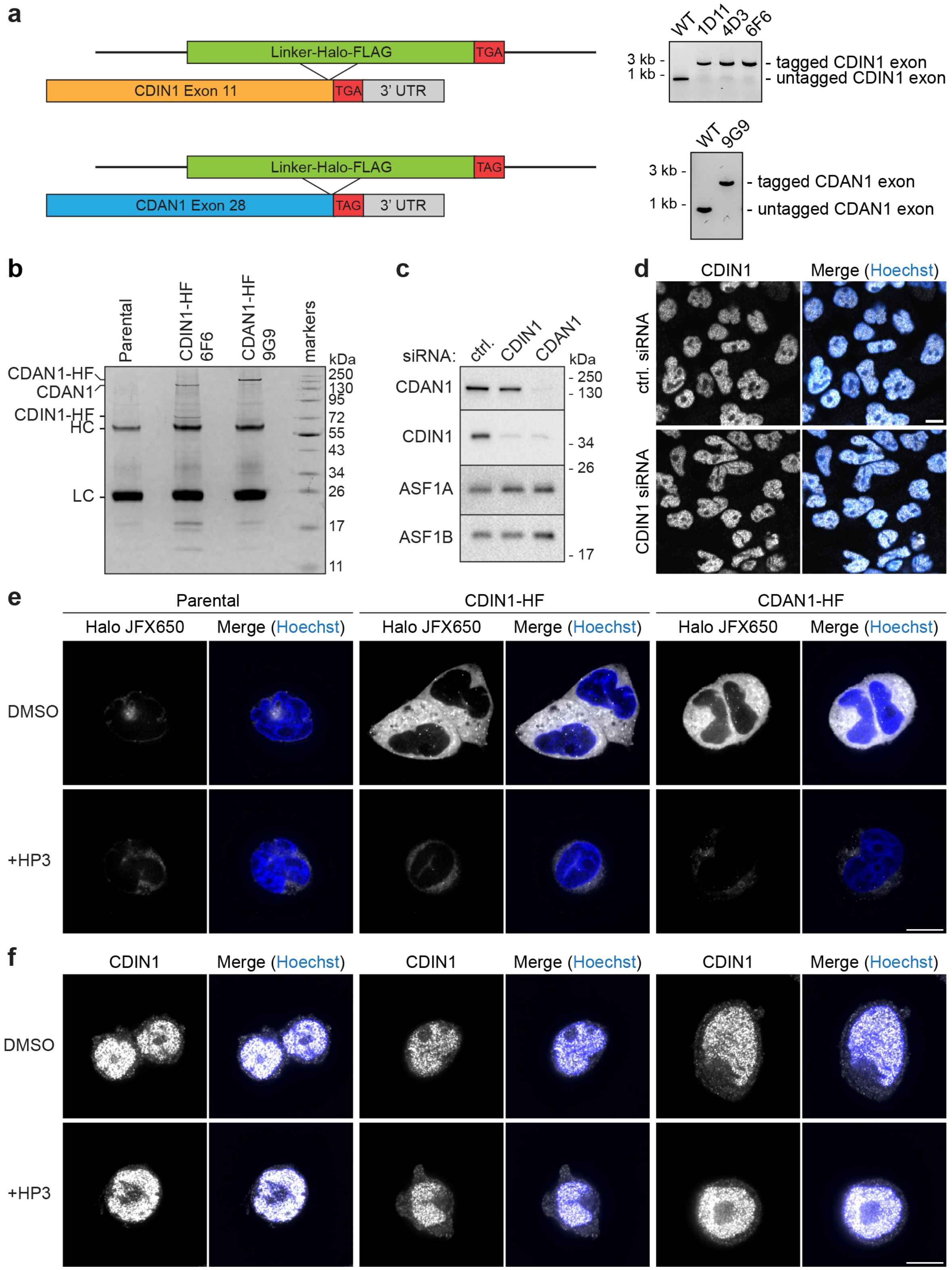

bioRxiv preprint doi: https://doi.org/10.1101/2024.08.08.607204; this version posted August 8, 2024. The copyright holder for this preprint (which was not certified by peer review) is the author/funder, who has granted bioRxiv a license to display the preprint in perpetuity. It is made available under aCC-BY 4.0 International license.

Supplemental Figure 1. Characterization of endogenous CDAN1 and CDIN1. a, Scheme (left) for endogenously tagging CDIN1 and CDAN1 with a C-terminal HaloTag-FLAG (HF) tag and PCR validation of knock-in clones (right). **b,** SDS-PAGE and Coomassie staining of anti-FLAG resins after immunoprecipitations of lysates from the indicated parental, CDIN1-HF, and CDAN1-HF Flp-In 293 T-REx cells. HC – heavy antibody chain, LC – light antibody chain. Note: CDIN1-HF copurifies an approximately equimolar amount of endogenous CDAN1; endogenous CDIN1 between LC and HC. **c,** SDS-PAGE and immunoblotting of lysates of Flp-In 293 T-REx cells treated with control (ctrl.) siRNAs or siRNAs against CDIN1 or CDAN1. Note: knocking down CDAN1 destabilizes CDIN1, but not vice versa; representative of 3 independent replicates. **d,** Immunofluorescence using anti-CDIN1 antibody of Flp-In 293 T-REx cells treated with ctrl. siRNA (siNeg) or siRNA against CDIN1, co-stained with Hoechst. Note: the nuclear signal observed with the anti-CDIN1 antibody does not decrease after knocking down CDIN1. **e,** Live-cell imaging of the indicated cells labeled with the JFX650 HaloTag ligand after treatment with DMSO or 500 nM HaloPROTAC3 (HP3) for 48 hr. Note: the specific cytosolic HaloTag signal in CDIN1-HF and CDAN1-HF cells is abolished after HP3 treatment. **f,** Immunofluorescence of cells from **e** after fixation using anti-CDIN1 antibody. Note: signal from the antibody is observed in all conditions and does not decrease in any cell line after HP3 treatment. Scale bar, 10 μm for all images.

bioRxiv preprint doi: https://doi.org/10.1101/2024.08.08.607204; this version posted August 8, 2024. The copyright holder for this preprint (which was not certified by peer review) is the author/funder, who has granted bioRxiv a license to display the preprint in perpetuity. It is made available under aCC-BY 4.0 International license.

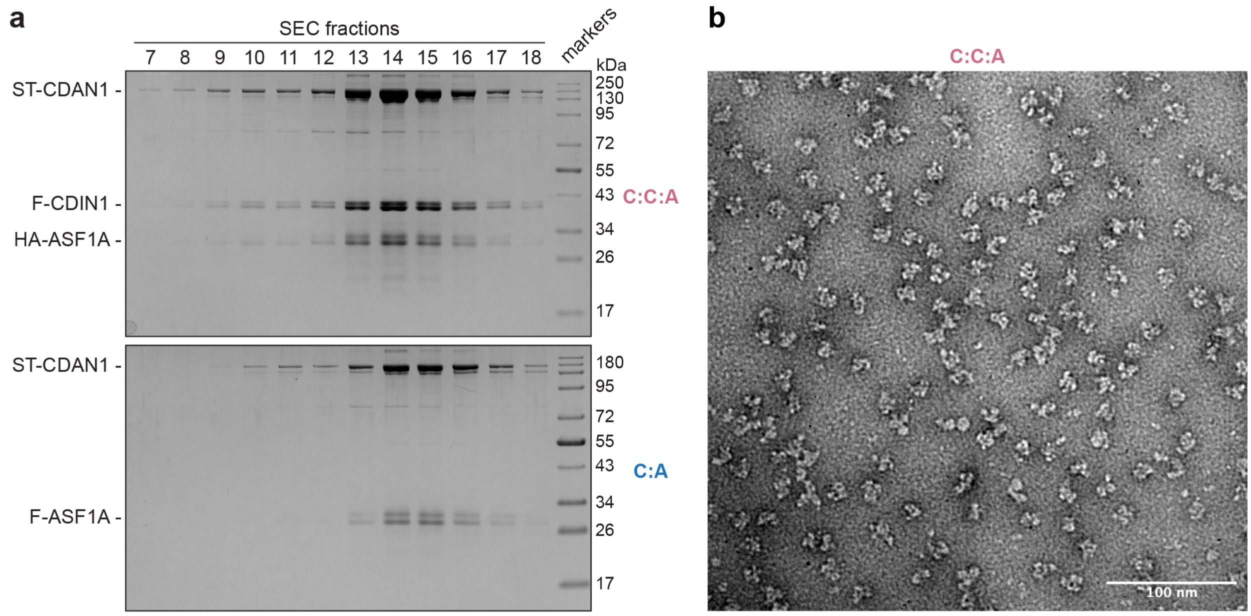

Supplemental Figure 2. Characterization of purified CDAN1 complexes. a, Size exclusion chromatography fractions of purified CDAN1-CDIN1-ASF1A (C:C:A; top) or CDAN1-ASF1A (C:A; bottom) analyzed by SDS-PAGE and Coomassie staining show comigration of the complex components. **b,** Representative negative stain EM image of the C:C:A complex.

bioRxiv preprint doi: https://doi.org/10.1101/2024.08.08.607204; this version posted August 8, 2024. The copyright holder for this preprint (which was not certified by peer review) is the author/funder, who has granted bioRxiv a license to display the preprint in perpetuity. It is made available under aCC-BY 4.0 International license.

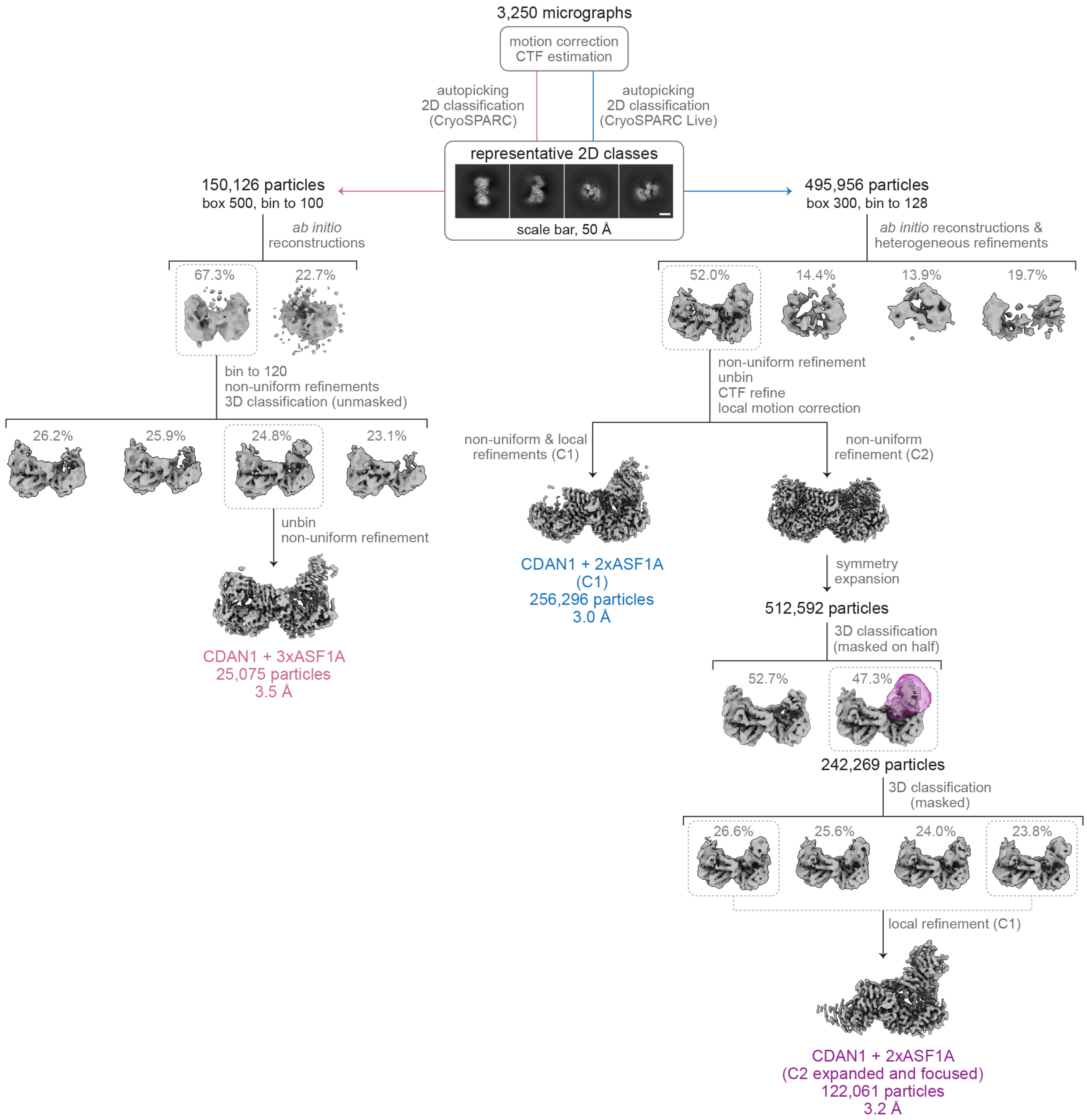

Supplemental Figure 3. Cryo-EM data processing summary. Overview of single-particle cryo-EM data processing and classification pipeline. The map with three ASF1A molecules was obtained from a separate autopicking and 2D classification pipeline using a larger box size (left) than the other maps, which were obtained from initial processing in CryoSPARC Live. Because asymmetry was consistently observed in the maps, the refinement with C2 symmetry was only used for symmetry expansion. The expanded particle set was then subjected to 3D classification masked on elements on one half of the map to obtain higher resolution insights into how two ASF1A molecules are stacked on one side of the CDAN1 dimer.

bioRxiv preprint doi: https://doi.org/10.1101/2024.08.08.607204; this version posted August 8, 2024. The copyright holder for this preprint (which was not certified by peer review) is the author/funder, who has granted bioRxiv a license to display the preprint in perpetuity. It is made available under aCC-BY 4.0 International license.

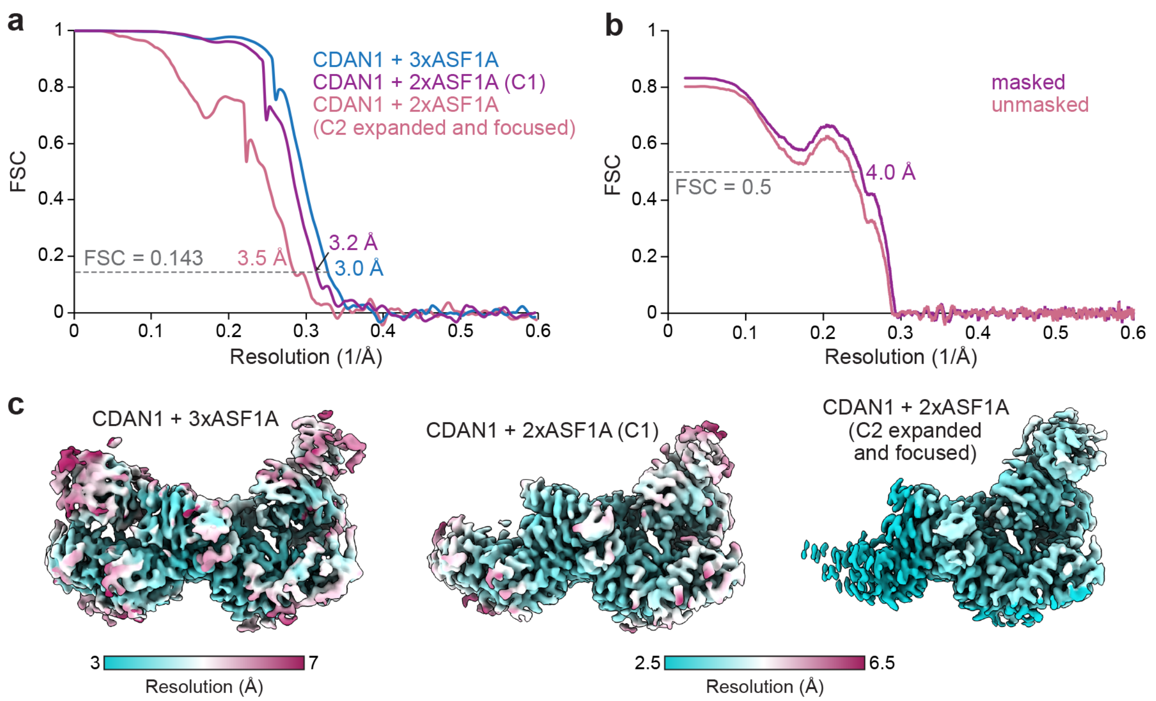

Supplemental Figure 4. Quality of maps and models. a, Fourier shell correlation (FSC) vs. resolution (1/Å) curves for the indicated cryo-EM maps. **b,** Model vs. map FSC curves. **c,** The indicated cryo-EM maps colored by local resolution.

bioRxiv preprint doi: https://doi.org/10.1101/2024.08.08.607204; this version posted August 8, 2024. The copyright holder for this preprint (which was not certified by peer review) is the author/funder, who has granted bioRxiv a license to display the preprint in perpetuity. It is made available under aCC-BY 4.0 International license.

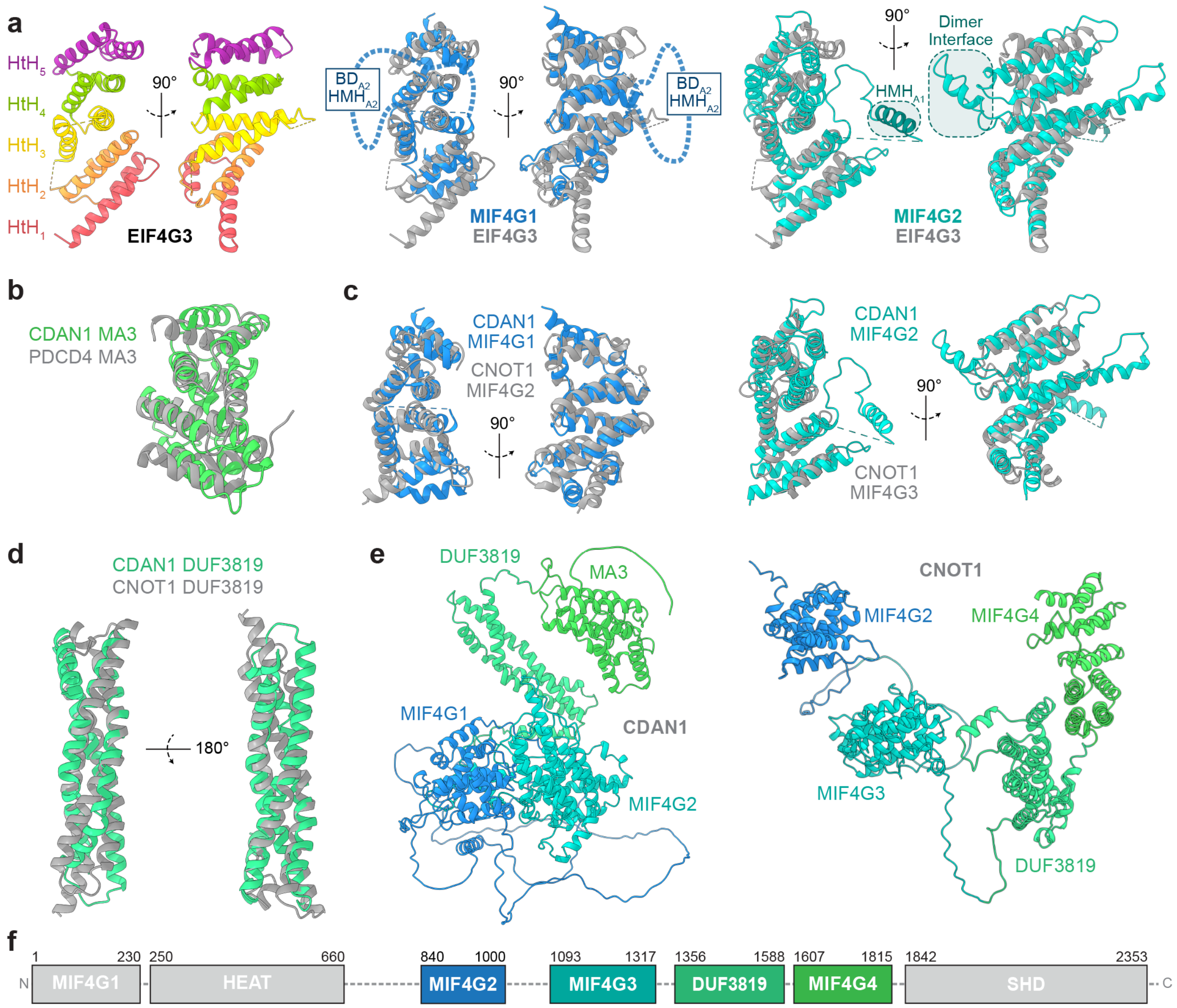

Supplemental Figure 5. CDAN1 domain analysis. a, Canonical MIF4G domain of eIF4G3 (PDB 1HU3) with each helix-turn-helix (HtH) motif colored separately (left) or superposed (gray) with CDAN1 MIF4G1 (middle; blue) or CDAN1 MIF4G2 (right; teal). **b,** Canonical MA3 domain of PDCD4 (PDB 2RG8, gray) superposed with the CDAN1 MA3 domain (green; Dali Z-score 7.8). **c,** Superposition of CDAN1 MIF4G1 (blue) with CNOT1 MIF4G2 (gray, left) or of CDAN1 MIF4G2 (teal) with CNOT1 MIF4G3 (gray, right). **d,** Superposition of the three-coil bundle domain of unknown function (DUF3819) predicted to be present in both CDAN1 (sea green) and CNOT1 (gray). **e,** Alphafold2 model of CDAN1 (left) or the central region of CNOT1 (right) colored by domain. **f,** Domain scheme of CNOT1 with the central region similar to CDAN1 colored as in **e**.

bioRxiv preprint doi: https://doi.org/10.1101/2024.08.08.607204; this version posted August 8, 2024. The copyright holder for this preprint (which was not certified by peer review) is the author/funder, who has granted bioRxiv a license to display the preprint in perpetuity. It is made available under aCC-BY 4.0 International license.

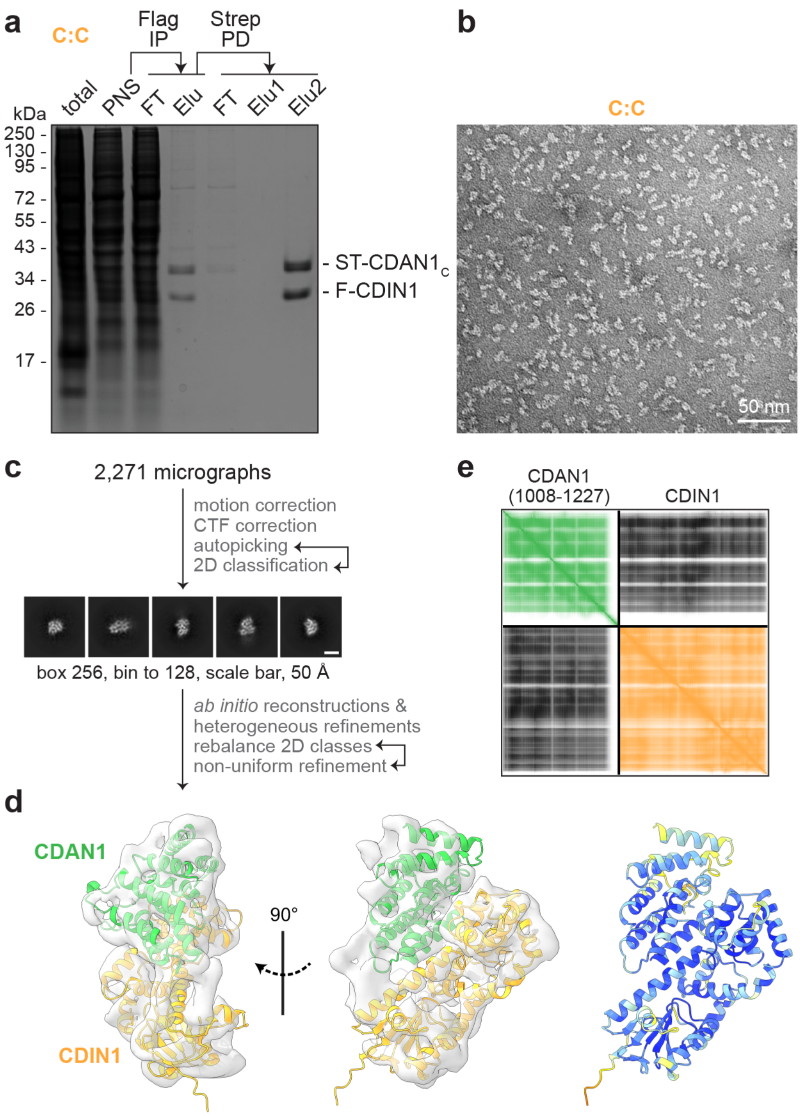

Supplemental Figure 6. Analysis of the CDAN1-CDIN1 interaction interface. a, The Strep-tagged MA3 domain of CDAN1 (ST-CDAN1$_C$;, residues 1008-1227) was co-expressed with FLAG-tagged CDIN1 (F-CDIN1) in Expi293 cells by transient transfection. The cells were then lysed, and the post-nuclear supernatants (PNS) were subjected to anti-FLAG immunoprecipitation (FLAG IP) followed by Strep-Tactin pulldowns (Strep PD) to purify the CDAN1$_C$-CDIN1 (C:C) complex. The total lysate, PNS, flow-through (FT), and elution (Elu) samples were analyzed by SDS-PAGE and Coomassie staining. **b,** Representative negative stain EM image of the C:C complex. **c,** Summary of cryo-EM data processing scheme for the C:C complex. **d,** Colabfold model of the C:C complex colored by chain (CDAN1 – green, CDIN1 – light orange) or pLDDT values docked into a ~6 Å cryo-EM map (left, transparent gray)**. e,** Predicted alignment error (PAE) plot of the Colabfold[50] prediction of the C:C complex.

bioRxiv preprint doi: https://doi.org/10.1101/2024.08.08.607204; this version posted August 8, 2024. The copyright holder for this preprint (which was not certified by peer review) is the author/funder, who has granted bioRxiv a license to display the preprint in perpetuity. It is made available under aCC-BY 4.0 International license.

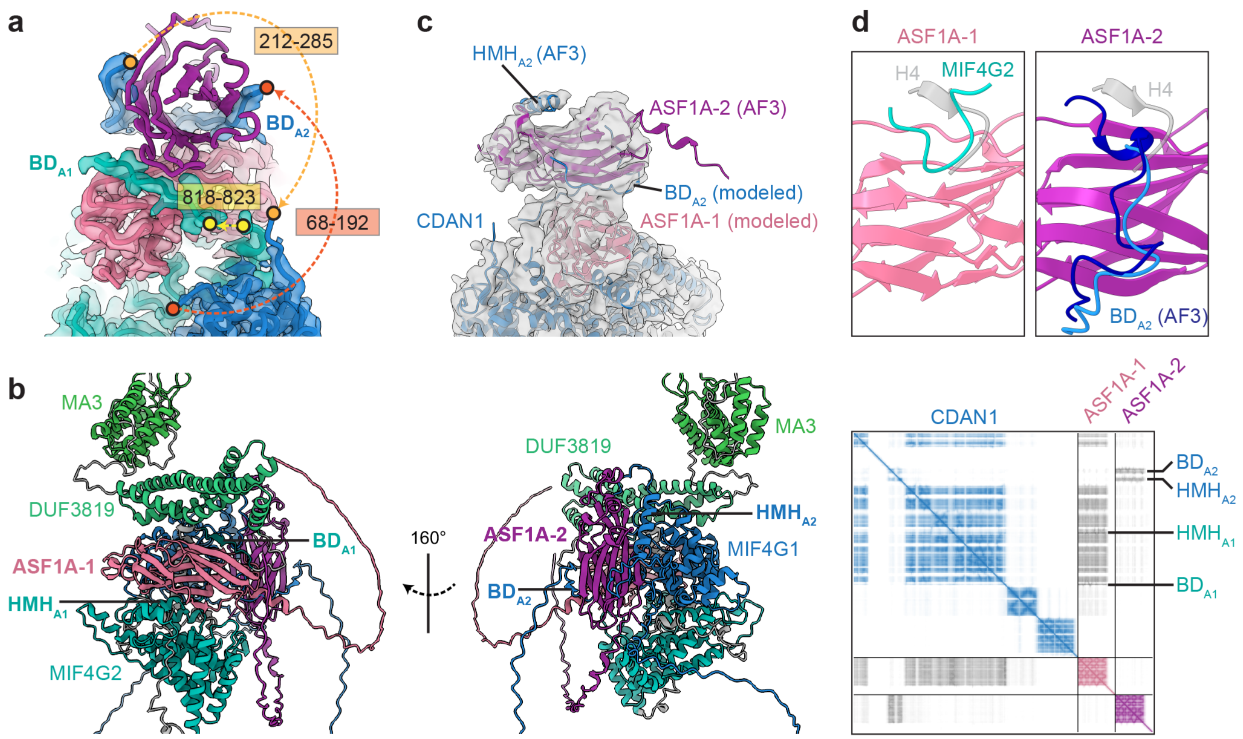

Supplemental Figure 7. CDAN1 interactions with ASF1A. a, CDAN1 B-domain assignments based on connectivity. Overlay of the structural model of CDAN1 bound to two stacked ASF1A molecules with the focused cryo-EM map contoured at 4.4σ; density for ASF1A-2 is omitted for clarity. A >200 amino acid loop (residues 69-284) extending from CDAN1 MIF4G2 (blue) harboring the previously identified B-domain sequence (BD_{A2}; residues 192-212 are modeled) and a putative H3 mimic helix (see **c**) is sufficiently long to engage the distal ASF1A-2 (purple). Dark orange and light orange dotted lines indicate how BD_{A2} links to the structured portion of MIF4G2. In contrast, the newly identified BD_{A1} sequence (residues 823-832) can only reach to ASF1A-1 (pink) from the modeled structured domain following MIF4G (yellow dotted line). **b,** Alphafold3 (AF3) model (left) and of CDAN1 with two ASF1A molecules, colored as in Fig. 3, and associated predicted alignment error (PAE) plot (right) predict distinct B-domain (BD) and H3 mimic helix (HMH) interactions with each ASF1A. **c,** The AF3 model of ASF1A bound to the putative HMH_{A2} spanning residues 247-260 of CDAN1 was superposed to ASF1A-2 of our structural model as in Fig. 3d and docked into the unsharpened CDAN1 + 3xASF1A map contoured at 6.8σ. Note: unmodeled density corresponding to the position of HMH_{A2} predicted by AF3. **d,** Structural models of ASF1A-1 and a CDAN1 MIF4G2 loop (residues 469-476; left) or the AF3 model of ASF1A-2 engaged with the extended BD_{A2} modeled in our cryo-EM structure (residues 201-212; light blue) or residues 201-218 (dark blue) in the AF3 model (right), both superposed with PDB 2IO5 showing the position of the C-terminal tail of histone H4 (residues 96-101; transparent gray). Note: clash of CDAN1 elements with the H4 tail position on both ASF1A-1 and ASF1A-2.

bioRxiv preprint doi: https://doi.org/10.1101/2024.08.08.607204; this version posted August 8, 2024. The copyright holder for this preprint (which was not certified by peer review) is the author/funder, who has granted bioRxiv a license to display the preprint in perpetuity. It is made available under aCC-BY 4.0 International license.

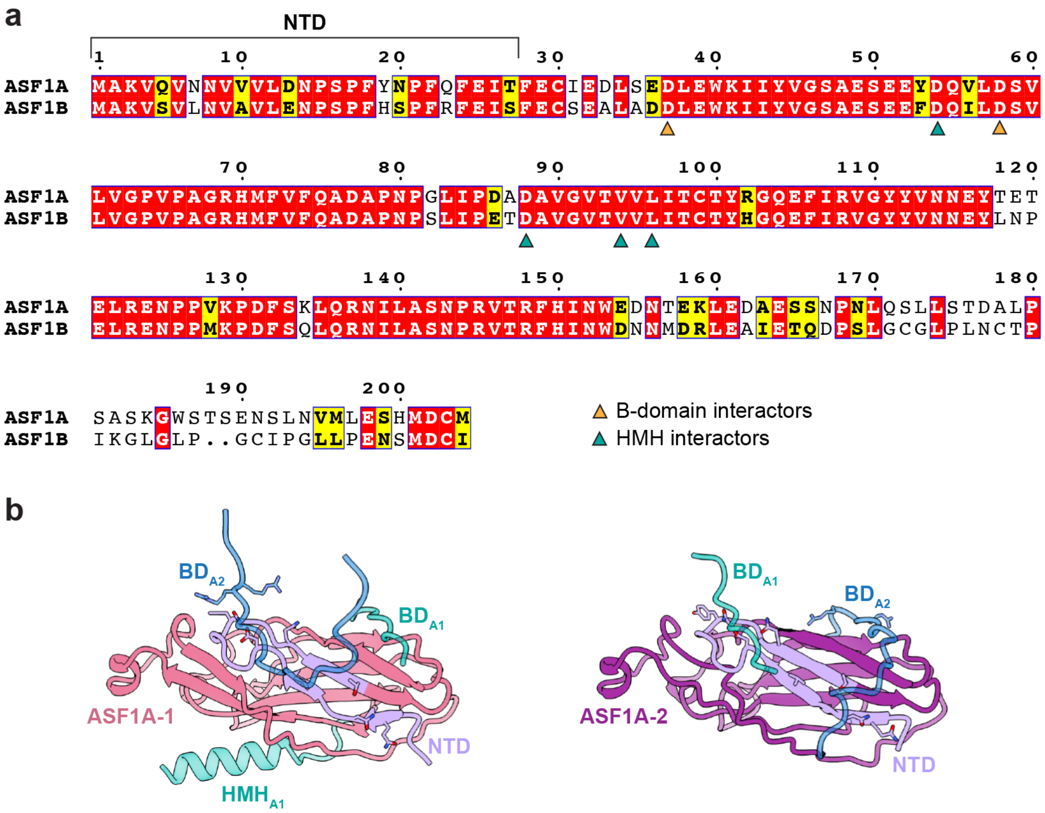

Supplemental Figure 8. ASF1A and ASF1B comparisons. a, Sequence alignment of ASF1A and ASF1B. Residues that mediate interaction with B-domains (orange triangle) and with histone H3 or H3 mimic helices (HMH; teal triangles). **b,** Placement of CDAN1 B-domains (BD) and the proximal HMH relative to ASF1A-1 (left) and ASF1A-2 (right). The N-terminal domain (NTD; lavender) on each ASF1A is indicated.
